# Adsorption of radon on silver exchanged zeolites at ambient temperatures

**DOI:** 10.1038/s41598-023-33253-5

**Published:** 2023-04-26

**Authors:** Stephan Heinitz, Jasper Mermans, Dominic Maertens, Hanna Skliarova, Alexander Aerts, Thomas Cardinaels, Christophe Gueibe, Jos Rutten, Natalie Ireland, Daniel Kuznicki, Steven Kuznicki

**Affiliations:** 1 Belgian Nuclear Research Centre (SCK CEN), Boeretang 200, 2400 Mol, Belgium; 2 Department of Chemistry, KU Leuven, Celestijnenlaan 200F, P.O. 2404, 3001 Leuven, Belgium; 3 Extraordinary Adsorbents Inc, 86 Greystone Crescent, Sherwood Park, AB T8A 3E6 Canada; 4 Department of Chemical and Materials Engineering, University of Alberta, Edmonton, AB T6G 2R3 Canada

**Keywords:** Environmental impact, Nuclear chemistry, Risk factors, Chemical engineering, Porous materials, Environmental monitoring

## Abstract

Since more than 100 years, the adsorption of the radioactive noble gas radon (^222^Rn) is performed on activated charcoal at cryogenic temperatures. There is little—if any—progress in the field of radon adsorption at ambient conditions to facilitate the development of simple and compact radon adsorption systems. We report here on the truly remarkable property of the synthetic silver-exchanged zeolites Ag-ETS-10 and Ag-ZSM-5 to strongly adsorb radon gas at room temperature. ^222^Rn breakthrough experiments in nitrogen carrier gas have shown that these materials exhibit radon adsorption coefficients exceeding 3000 m^3^/kg at 293 K, more than two orders of magnitude larger than any noble gas adsorbent known to date. Water vapor and carrier gas type were found to strongly influence radon adsorption, practically qualifying these silver exchanged materials as a new class of radon adsorbents. Our results demonstrate that Ag-ETS-10 and Ag-ZSM-5 are materials that show high affinity towards radon gas at ambient temperatures making them candidate materials for environmental and industrial ^222^Rn mitigation applications. Adsorption systems based on silver loaded zeolites have the potential to replace activated charcoal as material of choice in many radon related research areas by avoiding the necessity of cryogenic cooling.

## Introduction

Radon (^222^Rn, *T*_1/2_ = 3.8235 days) is a radioactive noble gas omnipresent in the environment. Being part of the ^238^U/^226^Ra decay chain, it has been found to considerably contribute to the radiation dose to the public^1^. Exposure to ^222^Rn at home and workplace is closely monitored in risk areas with high radon concentration and specific guidelines exist worldwide to mitigate exposure^2^. There is a reviving interest in ^226^Ra as raw material in the production of medical isotopes for cancer therapy that will require the handling of radiologically significant quantities of radon gas^3^. ^222^Rn has also gained considerable attention in particle physics research, where it is considered as unwanted contaminant in low-background environments^4^. There has been extensive research on ^222^Rn removal since the discovery of its adsorption on activated charcoal by Rutherford in 1906^5^ and numerous scientific publications exist on the adsorption properties of this material, including investigations on the influence of temperature, gas flow velocity, type of investigated charcoal etc.^6–8^. In the recent decades, additional materials have been proposed for ^222^Rn adsorption such as metal organic frameworks (MOFs)^9,10^, carbon molecular sieves (CMSs)^7^ and natural or synthetic zeolites^11^. However, most of these materials including activated charcoal were shown to exhibit adsorption coefficients below 10 m^3^/kg at room temperature and as of today, charcoal still remains the material of choice despite its limited radon retention characteristics at ambient temperatures^12^. Examples for radon reduction systems exist in underground laboratories, where ^222^Rn adsorption relies on cryogenic cooling of a charcoal bed involving large bed volumes^13,14^.

It is interesting to note that ongoing research at CEA, France, related to radioactive Xe capture for monitoring nuclear activities in the frame of the comprehensive nuclear-test-ban treaty as well as for reducing emissions from nuclear processing facilities, has considerably advanced towards the usage of synthetic zeolites for noble gas adsorption at room temperature. Xenon, being the homologue of radon, was found to be efficiently retained on materials such as Ag-ZSM-5 and Ag-ETS-10 and experimental evidence exists on the nature of strong adsorption sites majorly influencing the interaction of the noble gas with metallic silver nanoparticles^15,16^. Adsorption enthalpies for Xe on Ag-ZSM-5 were reported to be as high as 65 kJ/mol and remarkable findings have been reported for room temperature Xe capture and purification using adsorbent-supported silver nanoparticles^16,17^. Based on first test results^18^, there is strong evidence to assume that ^222^Rn will exhibit even stronger adsorption behavior towards these materials. While it was shown that radon has affinity to bind to metal surfaces^19^ and that silver in synthetic zeolites has a strong influence on the adsorption process^20,21^, an experimental determination of adsorption coefficients for ^222^Rn with a synthetic zeolite material containing silver has never been published in peer reviewed journals.

The ability to exchange a broad variety of cations into the framework of the Engelhard Titano-Silicate (ETS) and Zeolite Soconi Mobil (ZSM) structures has opened up a broad field of applications for these materials. Having a variety of different crystalline phases, synthetic zeolites such as ETS-4 or ZSM-5 have been investigated as catalytic reagents in the petroleum industry and separation chemistry^22–25^. Ag-ZSM-5 and Ag-ETS-10 are the silver exchanged variants of these materials known for exhibiting antibacterial properties^26^. Both forms are unusual in their ability to, upon heating, auto-reduce exchanged silver to nanospheres of dimensions of < 10 nm held on the zeolite surface at extraordinary large concentrations with essentially pure metallic character^27^. The usage of zeolites in the nuclear sector is especially interesting due to their radiation and fire resistance thanks to a rigid structure and inorganic nature. For example, the silver exchanged form of 13X zeolite (Ag-13X) has been studied as adsorbent for iodine^28^ and radon^20^ originating from processing of radioactive materials.

Since no publication exists on the interaction of Ag-ETS-10 and Ag-ZSM-5 with radon gas, a series of ^222^Rn adsorption experiments was performed at ambient temperature using these synthetic zeolites including Ag-13X and compared with two activated charcoals commonly encountered in radon adsorption.

## Comparison of ^222^Rn adsorbents

For the experimental evaluation of ^222^Rn adsorption on the studied zeolite materials, a dedicated system was designed in order to ensure data reproducibility. It was noted in the early stages of this study that different weather conditions influenced the obtained results by changes in relative humidity of the laboratory. Only an improved leak-tight setup enabling in-situ thermal regeneration of the studied materials resulted in satisfactory data quality and reproducibility.

**Figure 1:**
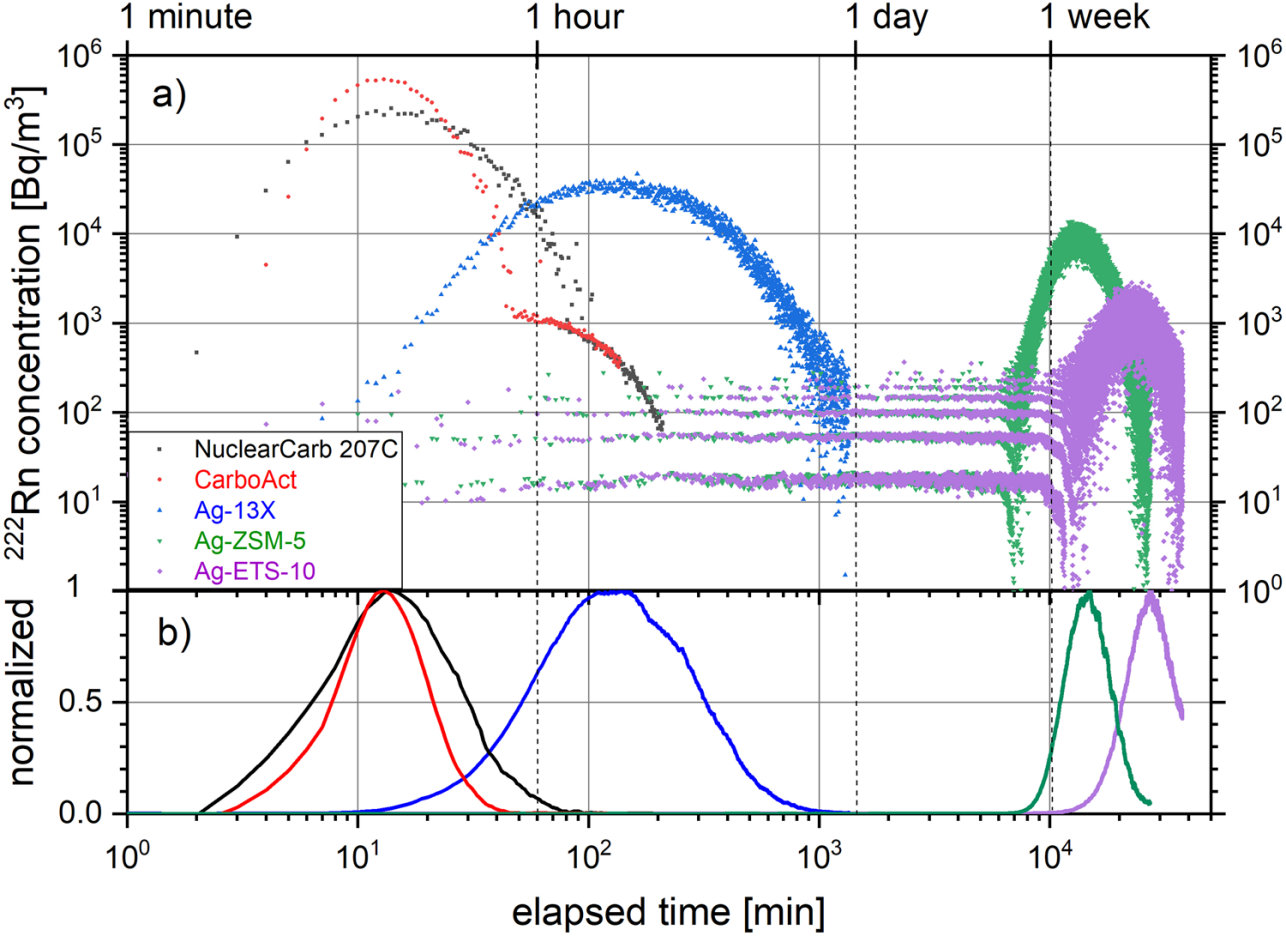
(**a**) Measured breakthrough curves of ^222^Rn on NuclearCarb 207C, CarboAct, Ag-13X, Ag-ZSM-5 and Ag-ETS-10 for an adsorbent bed volume of 17 cm^3^; temperature 293 K; N_2_ carrier gas flow rate 2 NL/min; (**b**) normalized, decay-corrected and adjacent-averaged representation of data in (**a**).

The experimentally measured breakthrough curves at ambient temperature on a 17 cm^3^ adsorption column for two activated carbons NuclearCarb 207C and CarboAct as well as three investigated silver-exchanged zeolites Ag-13X, Ag-ZSM-5 and Ag-ETS-10 are given in Fig. 1. While the breakthrough of ^222^Rn on both activated carbons occurred within several minutes after injection, its retention on Ag-ZSM-5 and on Ag-ETS-10 is considerably longer (note that both axes in Fig. 1 are given in logarithmic scale). For the last named material, measurable release of ^222^Rn was observed to begin 7 days after injection and a full breakthrough curve could not be obtained within 4 weeks. Only 1/32th of its initial activity could be detected in the ejected gas stream due to its radioactive decay along the adsorption bed. For Ag-13X we found a stronger retention of ^222^Rn compared to the activated carbons, although considerably less than for the other two zeolite structures. A summary of the mass of each adsorbent material, the injected and ejected ^222^Rn activity, the Rn retention time, the packing density and the measured adsorption coefficient *K* is given in Table 1. For both investigated charcoals, the measured adsorption coefficients for radon are of same magnitude as typically reported in literature^7^.

**Table 1:** Overview of experimental parameters, obtained retention times and adsorption coefficients for the five tested materials given in Fig. 1; for NuclearCarb 207C, CarboAct and Ag-13X the injected and released ^222^Rn activities do not significantly differ within their uncertainties (± 10% at a confidence level of 68%).

Material	Packing density [kg/m^3^]	Mass	Mean retention time	Adsorption coefficient *K*	Injected ^222^Rn activity	Released ^222^Rn activity
		[g]	[min]	[m^3^/kg]	[kBq]	[kBq]
NuclearCarb 207C	450	7.65	21	5.6	11.7	12.4
CarboAct	250	4.20	15	7.2	16.3	16.0
Ag-13X	970	16.50	240	29	16.3	20.8
Ag-ZSM-5	520	8.85	1.54·10^4^	3500	1030	132
Ag-ETS-10	950	16.30	2.76·10^4^	3400	1070	34.2

## Carrier gas influence

**Figure 2:**
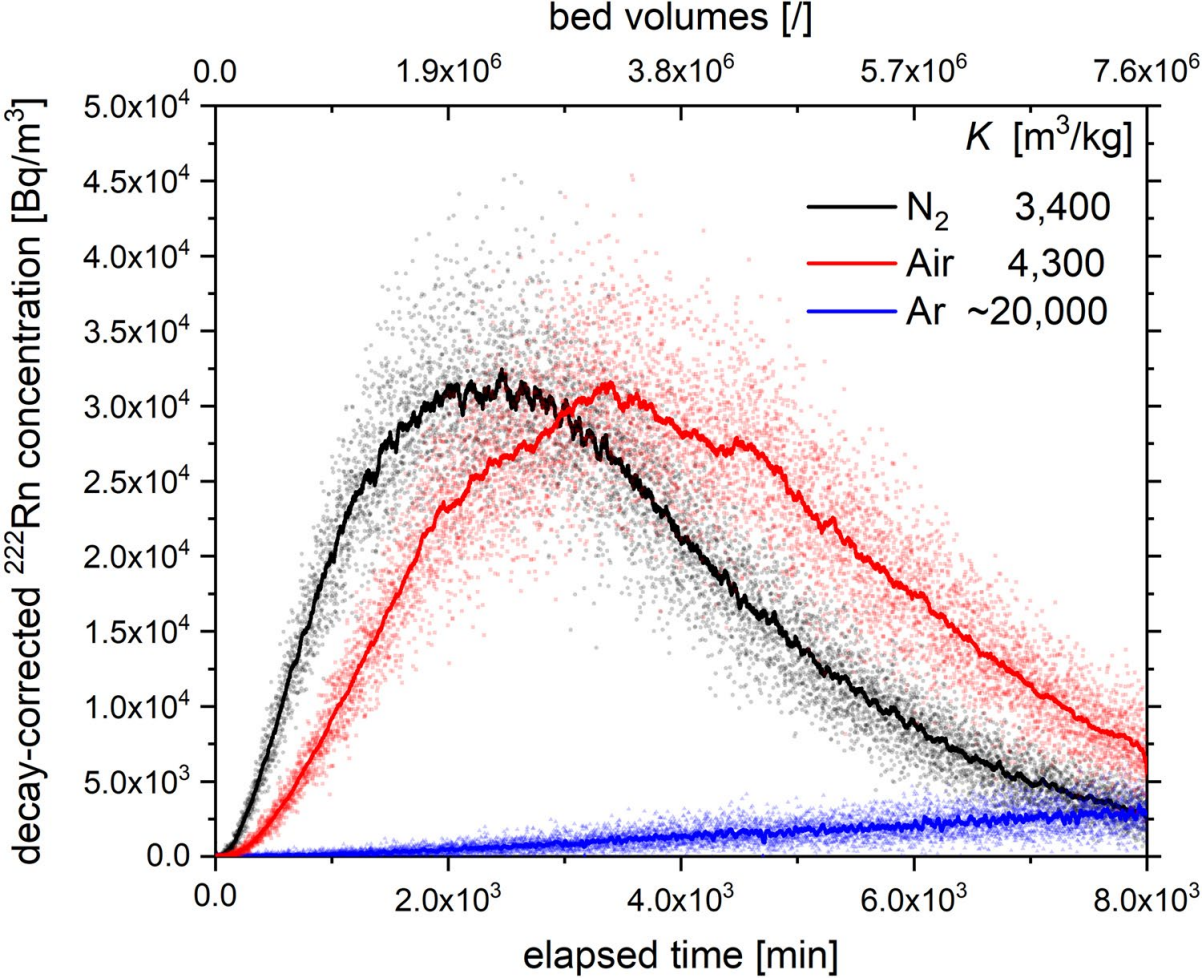
Decay-corrected experimental ambient temperature breakthrough curves of ^222^Rn with N5.0 N_2_, compressed air and bottled N4.8 Ar as carrier gases; Ag-ETS-10 adsorbent mass 3.0 g; volumetric flow rate 3 NL/min; injected ^222^Rn activity 485 kBq in each experiment.

Additionally, the influence of the carrier gas on the breakthrough of ^222^Rn has been measured on Ag-ETS-10 using 3.0 g of adsorbent at a flow rate of 3 NL/min to enable shorter experimental durations compared to experiments shown in Fig. 1. The experimental breakthrough curves in N_2_, compressed air and Ar are shown in Fig. 2. The results indicate a similar retention behavior of ^222^Rn in N_2_ and dry air, indicating that trace constituents of air that are not scavenged on the 13X-APG drying column have negligible adsorption competition with radon. Experimental ^222^Rn adsorption coefficients *K* were found to be 3400 and 4300 m^3^/kg for N_2_ and dry air, respectively. For Ar as carrier gas, however, no complete breakthrough curve could be obtained. Substantially stronger ^222^Rn retention on the adsorption bed was observed when compared to N_2_/air data, practically hindering the acquisition of a meaningful elution curve under the investigated conditions due to significant ^222^Rn decay loss on the adsorbent. Computational analysis indicated the ^222^Rn adsorption coefficient in Ar to be approx. a factor six larger than that in N_2_ at 293 K.

## Water competition

**Figure 3:**
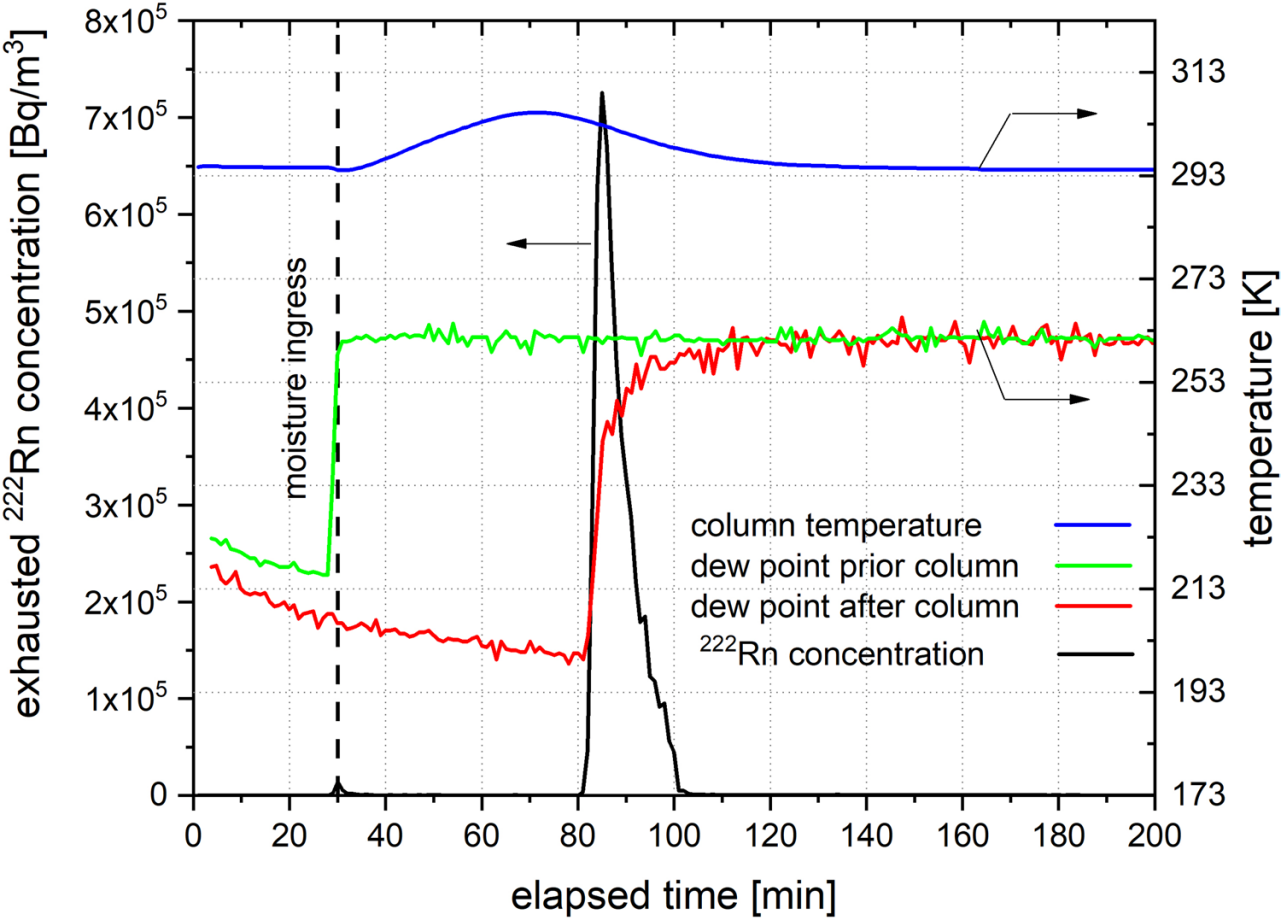
Measured release of ^222^Rn from Ag-ETS-10 upon contact with laboratory air of 55% rel. humidity at 294 K; flow rate 2 NL/min; mass of adsorbent—16.3 g; injected ^222^Rn activity—11.7 kBq; 30 min post injection the 13X dehumidification column was disconnected and laboratory air was allowed to enter the adsorption bed.

As described in the Materials and Methods section, all experiments were performed with pre-dried carrier gases to ensure low residual water content. The clear competitive effect of moisture towards ^222^Rn adsorption has been experimentally proven when laboratory air was allowed to pass over the adsorbent bed of Ag-ETS-10 loaded with ^222^Rn. Figure 3 gives the graphical representation of the ^222^Rn breakthrough that exactly coincides with the increase in dew point of the ejected air stream. A temperature increase of the adsorption bed is measured upon moisture entering the column indicating exothermic water adsorption. It can be seen that ^222^Rn leaves the adsorption bed as a sharply rising pulse, in contrary to the broad peak as observed in the previous experiments given in Figs. 1 and 2. This behavior can be explained by the competition of water molecules with ^222^Rn for the active adsorption sites on the adsorbent. Similar observations were made with Ag-ZSM-5. The subsequent thermal regeneration of the materials indicated that the desorption of ^222^Rn occurred quantitatively since no radon could be detected in the effluent gas stream. When saturated with water, Ag-ETS-10 and Ag-ZSM-5 showed negligible ^222^Rn retention compared to their regenerated form.

## Environmental radon capture

**Figure 4:**
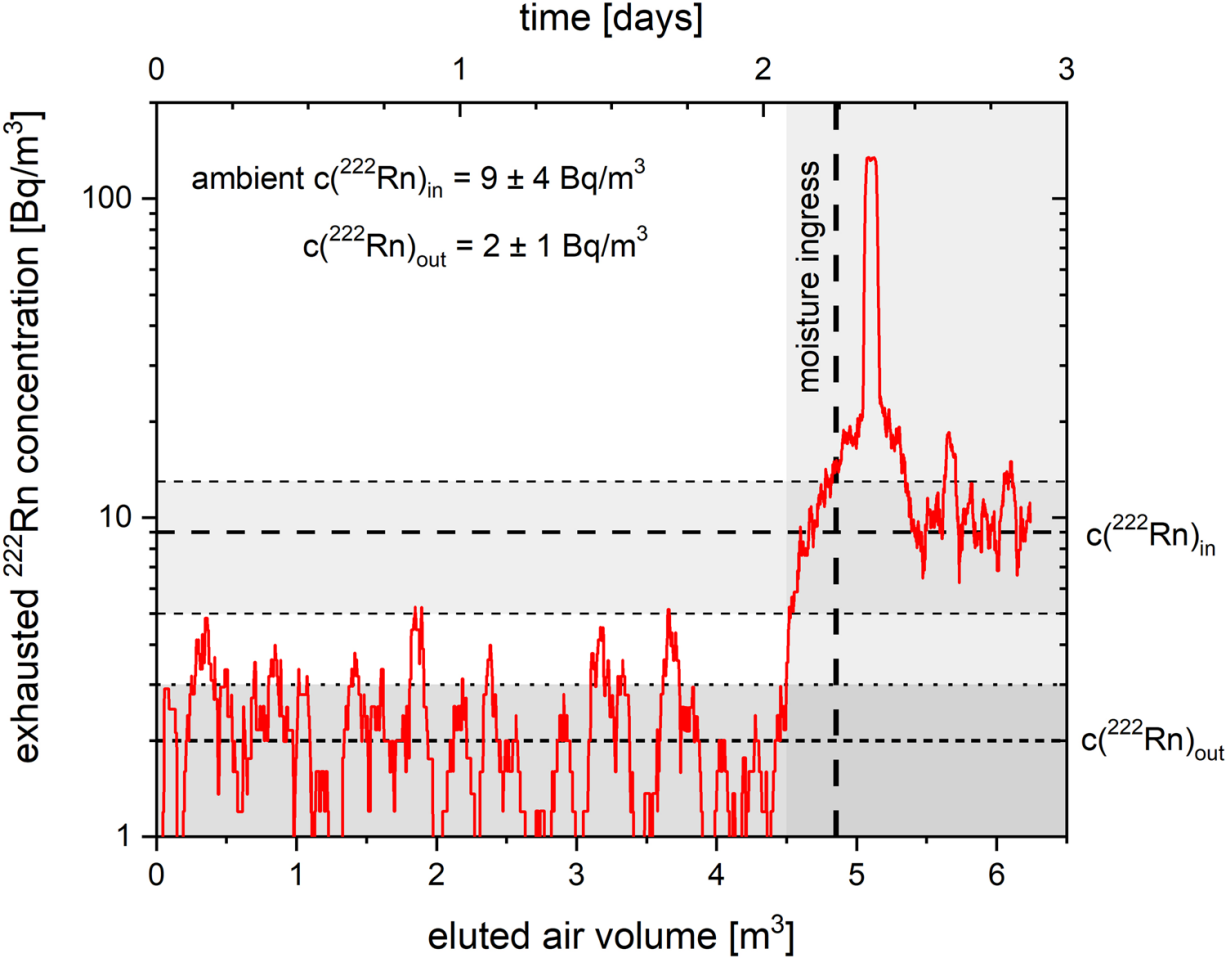
Measured ^222^Rn concentration in pre-dried laboratory air at 294 K after passing 16.2 g of Ag-ETS-10 at a flow rate of 1.5 NL/min; the 13X-4A dehumidification column was removed 2.25 d after start of the experiment; graph represents a 60 points adjacent average of collected data.

Laboratory air at 294 K with a ^222^Rn concentration ${C}_{in}^{Rn}$ of 9 ± 4 Bq/m^3^ and a relative humidity between 30 and 55% was pre-dried using 13X-4A and passed over a column containing 16.2 g of Ag-ETS-10. The measured ^222^Rn concentration in the exhausted air stream ${C}_{out}^{Rn}$ was 2 ± 1 Bq/m^3^, representing the lowest end in the measurement range of the employed radon detector. As seen in Fig. 4, these readings could be maintained for about 2 days, until a rise in radon concentration was noted and the pre-drying column subsequently removed similarly to the previous experiment represented in Fig. 3. A sharp increase in the measured ^222^Rn concentration was observed, indicating that ^222^Rn, which has accumulated on the Ag-ETS-10, was displaced by water molecules. Afterwards the measured radon concentration approached ${C}_{in}^{Rn}$ as would be expected for an adsorption bed that lost its radon retention property. Although suffering from in-leakage of moisture and radon into the adsorption bed and the employed radon detector operating in under-pressure, this experiment qualitatively shows the capability of the material to capture radon under environmental conditions.

## Discussion

Based on the measured breakthrough curves of ^222^Rn on the investigated materials, Ag-ETS-10 and Ag-ZSM-5 both showed remarkable retention properties towards radon at room temperature. For each of these two synthetic zeolite-type frameworks, substantial reduction of ^222^Rn in the effluent gas stream can be achieved. With 16.3 g of Ag-ETS-10 it was possible to retain 1 MBq of ^222^Rn in 2 NL/min N_2_ as carrier gas over a period of 1 week and more than 20 m^3^ (or 10^6^ bed volumes) of N_2_ have passed over the column before a measurable increase in ^222^Rn concentration could be detected at the exhaust. At maximum breakthrough, the radon release from the Ag-ETS-10 adsorption bed was delayed for 17 days, corresponding to approximately 4.5 half-lifes of ^222^Rn. With N_2_ as carrier gas, Ag-ZSM-5 and Ag-ETS-10 exhibit radon adsorption coefficients *K* that are at least two orders of magnitude larger compared to NuclearCarb 207C, CarboAct and Ag-13X. To our knowledge, no measurement has yet been reported in scientific literature showing a material with radon adsorption coefficients exceeding 3000 m^3^/kg at room temperature. It is also shown for Ag-ETS-10 that experiments with dry air resulted in 20% higher *K* values, whereas with argon only an estimation could be obtained due to substantially stronger ^222^Rn retention.

Moisture control was found to be of crucial importance for the evaluation of the obtained results. The investigated silver exchanged zeolites showed to be very potent desiccants and care must be taken interpreting ^222^Rn breakthrough experiments with regard to possible interference with moisture co-adsorption. Careful in-situ regeneration of the material avoiding exposure to ambient humidity has proven to be of high importance for data reproducibility. The competitive behavior of water towards ^222^Rn adsorption is similar to observations made with Xe adsorption on Ag-ETS-10^29^, and our results give evidence that the displacement of radon occurs quantitatively from the adsorption bed.

Non-polar gas species as noble gases have increasing polarizability with increasing electron shell radius^30^. This accounts for the increasing affinity of noble gases to strongly polarizing adsorption sites in the order Ar < Kr < Xe < Rn. In analogy, a similar behavior can be expected for diatomic molecules, where N_2_ shows higher polarizability if compared to Ar and O_2_^31^. Our experiments have shown that the choice of carrier gas had a significant influence on ^222^Rn adsorption since radon retention is majorly driven by competition with carrier gas molecules. A larger *K* value was measured for air (4300 kg/m^3^) than for pure N_2_ (3400 kg/m^3^), indicating that on macroscopic level, 21 vol.% of O_2_ in air induces a measurable difference in radon adsorption. For Ar, having a polarizability similar to that of O_2_, the radon adsorption coefficient on Ag-ETS-10 is approx. 6 times larger than for N_2_, comparable to the selectivity of N_2_ over Ar measured for this material^32^. It can be concluded that the physisorption of radon on the silver containing adsorption bed must be exceptionally strong when no carrier gas competition dominates. Changes in *K* values of this magnitude are not observed for activated charcoal at room temperature^33^, qualifying silver exchanged zeolites as a new class of radon adsorbents.

It is generally accepted in scientific literature that the interaction between xenon and the zeolite framework significantly enhances upon incorporation of silver^34,35^. It was speculated early that a substantial change in the electrostatic potential of the surface of the zeolite occurs upon loading with silver particles, leading to significantly enhanced noble gas affinity^34^. There is, however, no general consensus on what physico-chemical form of silver contributes to that effect. While some authors argue that adsorption in silver exchanged zeolites predominantly occurs via interaction with metallic silver nanopaticles^16,17^, others have recently reported evidence that the interaction is via Ag^+^ ions incorporated into the zeolite framework^34–37^. There is also no conclusive data to what extend the distribution and concentration of silver influence the adsorption properties towards noble gases. Since our results show that the obtained *K* values for Ag-ZSM-5 and Ag-ETS-10 do not significantly differ within their uncertainties, it appears that ^222^Rn retention does not primarily depend on the quantity of silver present when comparing Ag-exchanged zeolites to each other. This observation contradicts earlier findings made with Xe where adsorption capacity and the concentration of active adsorption sites was found to increase with larger silver loading^29,38^. While the ETS-10 framework is a titano-silicate with a wide pore structure able to exchange up to 30 wt% of silver, ZSM-5, being an alumino-silicate, has less cationic exchange sites to incorporate silver ions—only 10% by weight—and a smaller density. The latter material should thus have considerably less silver adsorption sites available for noble gases resulting in smaller *K* values—an observation that was not made in our experiments with radon. Moreover, Ag-13X, having similar Ag content and a density comparable to that of Ag-ETS-10, showed far inferior ^222^Rn retention properties giving rise to the conclusion that either the zeolite framework itself or in combination with the physical form, concentration and (size) distribution of silver defines the strength of noble gas interaction. It can be speculated that the procedure of preparing nano-dispersed silver onto the zeolite framework might have an influence on the noble gas adsorption behavior. Further investigations are necessary in order to fully understand the influence of material properties of silver exchanged zeolites towards noble gas adsorption.

The revolutionary nature of Ag-ETS-10 and Ag-ZSM-5 can be visualized by a following example of a Rn reduction system for air purification in laboratories requiring low background environments. For a certain radon inlet and outlet concentration ${C}_{in}^{Rn}$ and ${C}_{out}^{Rn}$, respectively, the ^222^Rn reduction factor (*RF*) is given by Eq. (1):

1 $$RF=\frac{{C}_{in}^{Rn}}{{C}_{out}^{Rn}}={e}^{\frac{\mathrm{ln}2\cdot K\cdot m}{f\cdot {T}_{1/2}^{Rn}}}$$

where *K* is the adsorption coefficient, *m* the mass of the adsorbent, *f* the volumetric flow rate and ${T}_{1/2}^{Rn}$ the half-life of the respective Rn isotope. For a constant inflow of ^222^Rn on an adsorption bed, a significant accumulation of ^222^Rn occurs on the column upon reaching steady-state conditions where the decay rate of adsorbed ^222^Rn approaches its incoming flux. Assuming a throughput of *f* = 100 m^3^/h of air with a ^222^Rn activity concentration of ${C}_{in}^{Rn}$ = 20 Bq/m^3^, an adsorption bed of 20 cm diameter and 50 cm height (*m* = 15 kg) could delay the incoming ^222^Rn stream by 600 h or 25 days (*K* = 4000 m^3^/kg). This would correspond to a breakthrough ^222^Rn activity concentration of ${C}_{out}^{Rn}$ = 0.22 Bq/m^3^ in the effluent air stream of a single continuously working adsorption column at room temperature. While similar adsorption systems based on activated charcoal require several cubic meters of bed volume and cryogenic cooling, this example clearly shows the economic potential of silver exchanged zeolites in ^222^Rn related research and gas separation applications. As results in Fig. 4 indicate, radon can be then efficiently stripped in a limited volume by allowing moisture to pass over the adsorption bed. Smaller scale radon reduction systems could be employed to reduce indoor ^222^Rn levels in dwellings located in elevated radon risk areas.

In contrast to adsorption beds based on charcoal, silver exchanged zeolite beds require periodic thermal regeneration in order to restore their initial ^222^Rn retention performance. Although it has been reported earlier that ionizing radiation might be the cause for the deactivation of Ag-13X^20^, we have strong reason to believe that primarily gaseous water co-adsorption is responsible for this effect by blocking the active sites of the adsorbent. This is supported by the fact that Ag-ETS-10 practically loses its radon retention properties if exposed to humidity levels typically present in the environment. Therefore, it needs to be clearly stated that proper removal of water using known dehumidification processes to achieve dew points below 200 K is crucial for the efficient operation of silver exchanged zeolite radon adsorbents. The usage of commonly employed gas drying agents such as 13X or 4A zeolites have proven to be practical for this purpose. Additionally, based on the results presented in Fig. 4, it can be concluded that proper in-situ regeneration of the adsorption material in leak-proof equipment is advisable to avoid moisture interference since a significant drop in ^222^Rn retention might be expected upon moisture co-adsorption.

Apart from water vapor, recent investigations also indicate that organic species containing chlorine show a measurable poisoning effect on Ag-ZSM-5 with regard to Xe adsorption^39^. It can be concluded that proper protection of the adsorption bed from species attacking the active sorption sites will be required. Yet another peculiarity that comes with the usage of these materials is the economic value of silver, making Ag-exchanged zeolites significantly more costly than activated carbon (5000 EUR/kg as of 2022). Due to their cost, the durability towards long term usage and multiple regeneration cycles has to be experimentally proven. Although first promising experience in studies with xenon exists^29^, it is important to verify physical and chemical adsorbent stability in representative environmental conditions and to understand the behavior towards trace constituents of air typically present in much higher atmospheric concentrations than ^222^Rn.

Simply based on polarizability considerations, fluorinated greenhouse gases such as chlorofluorocarbons (CFCs) or SF_6_ should exhibit significantly stronger retention on Ag-ETS-10 and Ag-ZSM-5 if compared to charcoal. Based on results for radon interaction with polycrystalline metal surfaces^19^, ETS-10 and ZSM-5 frameworks containing stable metal nanoparticles other than silver should exhibit even larger affinity towards ^222^Rn than reported herein. The result of this study should motivate further detailed investigations of metal exchanged zeolites towards novel developments in radon and other gas separation applications.

In conclusion, our results demonstrate exceptionally strong interaction of radon with silver exchanged ETS-10 and ZSM-5 frameworks, analogous to observations published earlier on xenon^17^. We have shown that moisture and the polarizability of the carrier gas have a strong influence on the adsorption behavior of ^222^Rn, practically making these silver exchanged zeolites a new family of radon adsorbents. More compact and simple ^222^Rn adsorption systems can be designed using these zeolite materials, considerably reducing the impact of ^222^Rn originating from ^226^Ra emanations in environmental, scientific and industrial applications.

## Materials and methods

### Materials

Ag-ETS-10 (granular, binderless, 16 × 30 mesh, packing density 950 kg/m^3^) was prepared and provided by Extraordinary Adsorbents Inc. of Edmonton, Alberta, Canada. Ag-13X (spherical, + 20 mesh, packing density of 970 kg/m^3^) was obtained from Sigma-Aldrich, Belgium. Nuclearcarb 207C (granular, 6 × 12 mesh, packing density of 450 kg/m^3^) was obtained from Chemviron Carbon, United Kingdom, and CarboAct High Purity Carbon (fragmented, 0.01–0.4 cm, packing density of 250 kg/m^3^) was obtained from Carbo-act International, Netherlands. Ag-ZSM-5 (spherical, particle size 0.2–0.5 mm, packing density of 520 kg/m^3^) was kindly provided by CEA, France, and was synthetized by Ag exchange of Na-PZ-2/25 obtained from Zeolyst, USA, according to the description given in the thesis of Deliere^18^. The moisture adsorbent 13X-APG was obtained from UOP CH Sarl, Switzerland, as spherical 8 × 12 mesh beads.

^222^Rn was generated and supplied by degassing a PYLON RN-1025 flow-through 3.77 MBq ^226^Ra source (reference date: 25/04/2014) supplied by PYLON Electronics Inc., Canada. Nitrogen was supplied in N5.0 purity from an in-house LN_2_ supply tank with a nominal pressure of 8 bar and a dew point of 203 K. Compressed air was supplied from an in-house air compressing station at 6 bar with a dew point of 243 K. Technical grade N4.8 argon was supplied in bottles by Air Products NV, Belgium.

### Methods

#### Synthesis of Ag-ETS-10

ETS-10 was synthesized by the suspension of particulates of anatase (TiO_2_) in a sodium silicate solution (~ 30% SiO_2_, 8.5% Na_2_O). After the addition of ETS-10 seeds and a fluoride mineralizer, the mixture was crystallized for 72 h at 473 K in a 3.8 L Parr stainless steel autoclave at autogenous pressure. After filtering, washing and drying, the crystalline product underwent granulation and was sieved into 16 × 30 mesh aggregates. The high purity, as synthesized, ETS-10 was loaded into a column and strip exchanged with an excess of silver nitrate in solution at 363 K for 36 h. The silver-exchanged ETS-10 (now Ag-ETS-10) was thoroughly washed with deionized water and dried at 473 K in a forced air oven.

#### Experimental Rn adsorption setup

**Figure 5:**
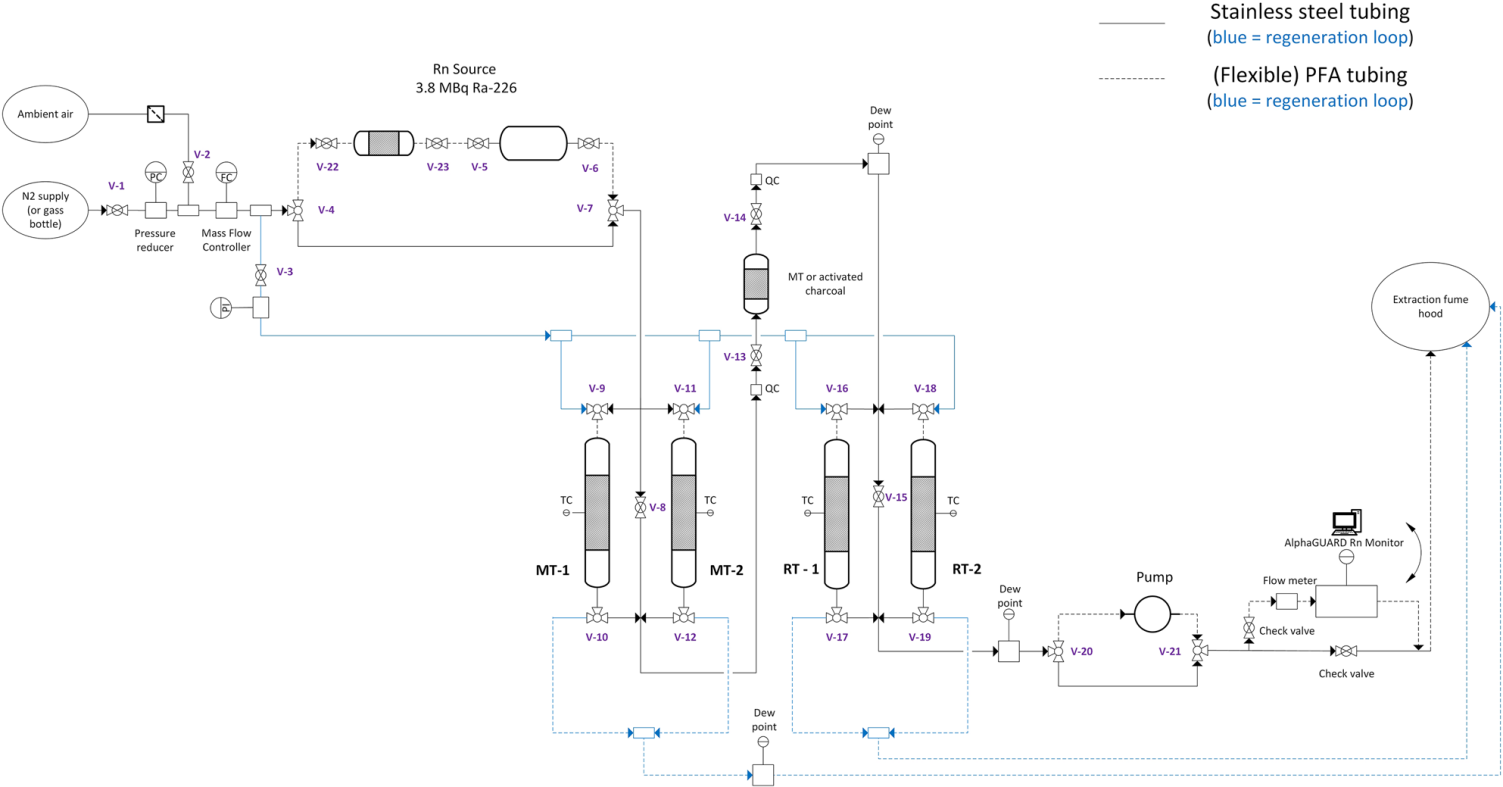
Schematic representation of the setup used for ^222^Rn adsorption experiments.

Dynamic ^222^Rn adsorption experiments were performed in a laboratory scale setup schematically depicted in Fig. 5. The trapping device consisted of two redundant moisture traps and two Rn adsorption columns, hygrometers for indication of the moisture content in the system, flow controllers (MFC and VA-flowmeter), a vacuum pump and a radon monitor. The system was built out of stainless steel fittings, valves and tubing (1/4″ OD) to assure leak tightness for minimal moisture ingress. A part of the system contained flexible perfluoroalkoxy alkane (PFA) tubing to ease operation.

Supplied N_2_, air and Ar was reduced in pressure and fed into the experimental system as carrier gas. The flow of the carrier gas in the setup was controlled by a thermal mass flow controller (Red-y smart, Vögtlin Instruments GmbH, Switzerland). The setup was equipped with two redundant moisture traps (MT-1 and MT-2) filled with molecular sieve 13X-APG to reduce the dew point of the incoming gas below 200 K. Redundancy was provided for continuous operation in case of column saturation. Both columns have a volume of approx. 385 cm^3^ (Ø_in_ = 4.05 cm, L = 30 cm) and are made of stainless steel (SS 316). The radon adsorption was performed with one of the adsorption columns RT-1 and RT-2, both having a volume of 17 cm^3^ (Ø_in_ = 1.2 cm, L = 15 cm) and made of stainless steel (SS 316). All columns are designed and manufactured in-house. For all columns, a CF flange with a copper seal on top of the column is installed to ensure leak tightness. The seal is regularly exchanged and a leak test is performed after each column opening via pressurization. Two dew point transmitters (Easidew online, Michell Instruments, United Kingdom, range between 173 and 293 K) are installed in the setup to measure the dew point of the carrier gas at the exhaust of MT-1/2 and RT-1/2, respectively.

The setup enables regeneration of different sorbents in situ, i.e. while keeping the sorbent in the column. The heating of the stainless steel column was performed via an electrical heating wire connected to a temperature controller that regulates the temperature to a predetermined set-point via a thermocouple. The thermocouple was positioned on the outer wall of the column. The columns and heating wire are thermally insulated with glass fiber tape. The setup was equipped with a vacuum pressure transmitter (pressure range − 1 to 2.5 barg) for pressure monitoring and leak tightness validation of the setup and the columns in particular after opening and closing.

A (removable) adsorbent column (120 cm^3^) can be inserted into the experimental loop via quick-connects to enable the degassing of the ^222^Rn source. Once removed, the loop was closed via stainless steel tubing. The outgoing radon concentration was monitored by sampling a part of the exhaust stream with a AlphaGUARD Professional Radon Monitor DF2000, Bertin GmbH, Germany. It enables a continuous determination of the volumetric radon concentration with a measuring range from 2 Bq/m^3^ to 2 MBq/m^3^ with a data sampling frequency of 1 min. The monitor was equipped with a flow-regulated pump which can be adjusted from 0.05 L/min to 2 L/min, allowing sampling of the exhausted gas stream. For the experiments described herein, the device was operated in the “1 min, flow, radon” mode at a flow rate of 1 L/min.

Experiments for environmental radon adsorption were performed in a non-radioactive laboratory at room temperature by using a KNF Laboport N86 vacuum pump and a mass flow controller (Red-y smart, Vögtlin Instruments GmbH, Switzerland) in suction mode through one MT400-4 and one MT120-4 13X-4A moisture traps (both obtained from Agilent, USA) and a glass column (Ø_in_ = 1.2 cm, L = 15 cm manufactured at Glasatelier Saillard, Belgium) containing the tested adsorbent. All components were connected by flexible PFA tubing. The exhausted air stream was sampled using a newly purchased AlphaGUARD DF2000 radon monitor having a negligible intrinsic background count rate.

#### Breakthrough experiments

Breakthrough experiments were performed at laboratory room temperature, which was 293 ± 2 K. A stepwise procedure was applied. At first, the Pylon RN-1025 was degassed to remove accumulated ^222^Rn in equilibrium with 3.77 MBq ^226^Ra. In order to control the total ^222^Rn activity injected in each performed experiment, the degassing was performed with dried laboratory air for 1 h at a flow rate of 0.5 NL/min, where the flushed ^222^Rn was adsorbed on a 50 g NuclearCarb 207C column that was subsequently removed. After degassing, radon started accumulating again in the RN-1025 source at a rate of 473 Bq/min. In a next step, the accumulated radon was injected into the selected adsorbent column by flushing the radon source with the desired carrier gas (N_2_, Ar or air) and flow rate (0.5–5 NL/min) for 5 min. In order to reach the desired activity for injection, the time between the end of the degassing and the end of the injection was adjusted, being 25–35 min for experiments with the activated carbons and Ag-13X and 18–40 h for Ag-ZSM-5 and Ag-ETS-10, respectively. After injection, the radon source was closed and N_2_, Ar or air was fed to the column until all injected radon has passed through the column (complete breakthrough) or the experiment terminated preliminarily (incomplete breakthrough).

#### Material regeneration

Each tested adsorbent underwent a regeneration cycle via heat treatment under inert gas conditions prior to each experiment. In each case, N5.0 nitrogen was used as regeneration gas. The regeneration was performed for at least 15 h at 503 K for Ag-ETS-10, 523 K for Ag-13X and Ag-ZSM-5, while for both activated carbons 423 K was chosen. The moisture adsorbent 13X-APG was regenerated at 553 K. The regeneration of 13X-4A and Ag-ETS-10 for environmental ^222^Rn adsorption was performed in a muffle furnace (type AAF 11/7, Carbolite, UK) at 503 K overnight and each material was subsequently filled manually into the respective adsorption column.

#### Data treatment

**Figure 6:**
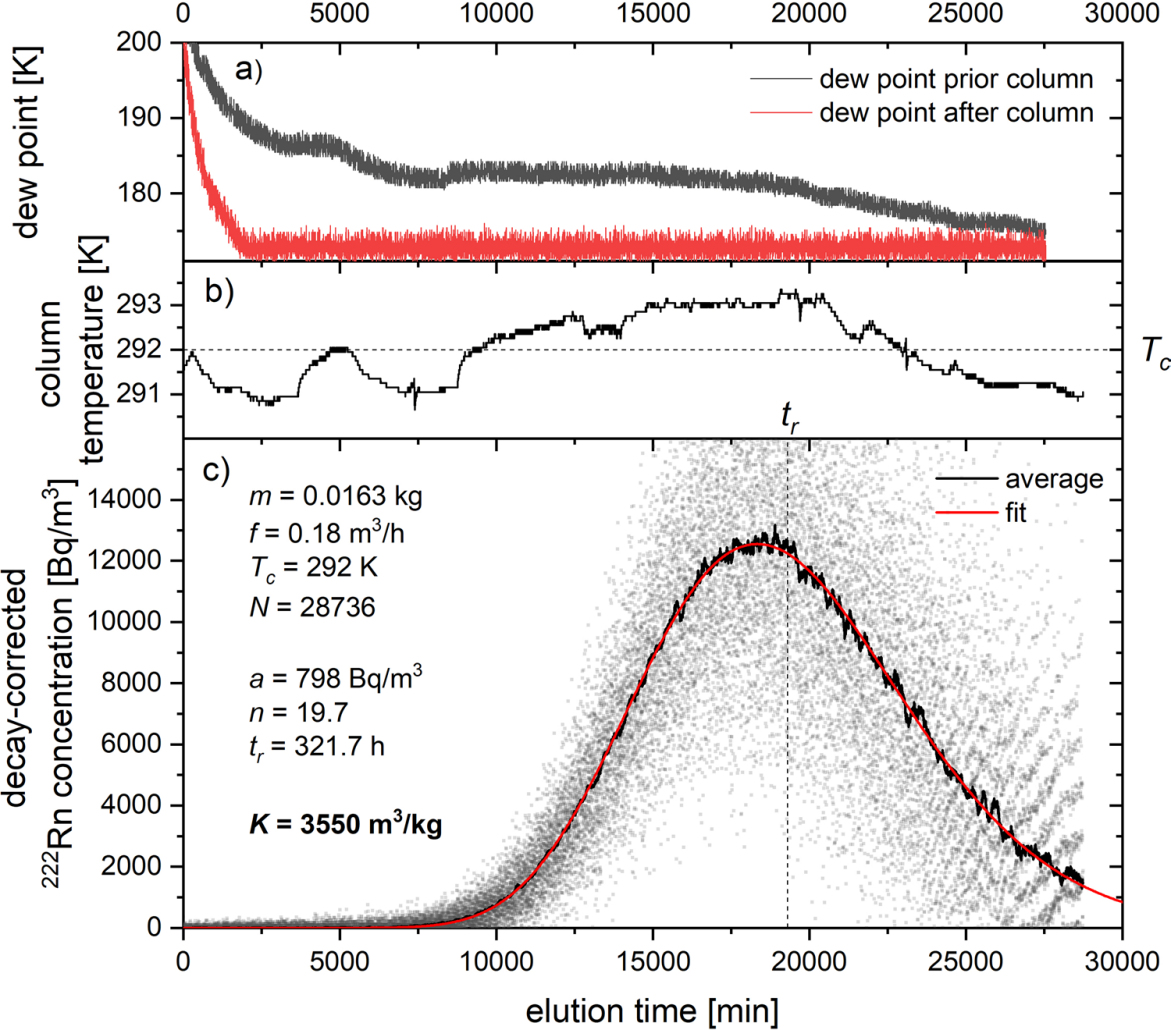
Experimental data for a breakthrough experiment with 16.3 g Ag-ETS-10 at a flow rate of 3 NL/min of N_2_ showing the evolution of (**a**) the dew point prior and after the adsorption column, (**b**) the column temperature and (**c**) the averaged decay-corrected ^222^Rn concentration with its plate model fit.

For each performed experiment, the data acquisition system of the AlphaGUARD DF2000 radon monitor provided the volumetric ^222^Rn activity concentration of the ejected gas stream as function of experimental time. These data were first corrected for the intrinsic background of the device, which was periodically determined by flushing pure N_2_ through the system and ranged typically between 20 and 100 Bq/m^3^. Afterwards, decay correction was applied to the background corrected dataset to account for ^222^Rn decay since the start of each breakthrough experiment. For each experimental data set of *N* measurement points, the breakthrough curves were then smoothed via the adjacent averaging method over $\sqrt{N}$ points. The adsorption coefficient *K* [m^3^/kg] for each tested adsorbent was calculated using the formula given as

$$K=\frac{{t}_{r}\cdot f}{m}$$

where *t*_*r*_ is the mean retention time in [h], *m* the mass of adsorbent in [kg] and *f* the volumetric flow rate in [m^3^/h]. The retention time *t*_*r*_ is the time of the eluted decay-corrected radon activity to be 50% of the total injected radon activity. Fitting of the breakthrough curves and the subsequent estimation of *t*_*r*_ was performed using software data analysis applying the chromatographic plate model function^6^ given as

$${C}_{out}^{Rn}\left(\frac{t}{{t}_{r}}\right)=\frac{a\cdot {n}^{n}}{\left(n-1\right)!}\cdot {\left(\frac{t}{{t}_{r}}\right)}^{n-1}\cdot {e}^{-\frac{nt}{{t}_{r}}}$$

where *a* is the amplitude of the injected ^222^Rn spike and *n* the number of theoretical plates of the adsorbent column. The model also served as computational analysis tool to estimate *t*_*r*_ for incomplete break-through curves by fitting the expected total ejected ^222^Rn activity to the activity initially injected into the column. An example of a fitting curve with the obtained plate model parameters is given in Fig. 6 together with data on temperature and dew point evolution for a typical breakthrough experiment.

The uncertainties for the adsorbent mass *m* and volumetric flow rate *f* were given as 1% and 2%, respectively. Variations in column temperature during experiments induced the largest uncertainty in the determination of *t*_*r*_. The overall uncertainty associated to the measured adsorption coefficient *K* was estimated to be in the order of 10% at a confidence level of 68%.

## Data Availability

The datasets used and analyzed during the current study are available from the corresponding author on request.
